# Alterations of the volatile metabolome in mouse models of Alzheimer’s disease

**DOI:** 10.1038/srep19495

**Published:** 2016-01-14

**Authors:** Bruce A. Kimball, Donald A. Wilson, Daniel W. Wesson

**Affiliations:** 1 United States Department of Agriculture, Animal and Plant Health Inspection Service, Wildlife Services, National Wildlife Research Center, Monell Chemical Senses Center, Philadelphia, PA 19104; 2 Emotional Brain Institute, Nathan S. Kline Institute for Psychiatric Research, Orangeburg, NY, 10962; 3 Department of Child & Adolescent Psychiatry, New York University School of Medicine, New York, NY, 10016; 4 Department of Neurosciences, Case Western Reserve University School of Medicine, Cleveland, OH 44106; 5 Department of Biology, Case Western Reserve University, Cleveland, OH 44106.

## Abstract

In the present study, we tested whether the volatile metabolome was altered by mutations of the Alzheimer’s disease (AD)-implicated amyloid precursor protein gene (*APP*) and comprehensively examined urinary volatiles that may potentially serve as candidate biomarkers of AD. Establishing additional biomarkers in screening populations for AD will provide enhanced diagnostic specificity and will be critical in evaluating disease-modifying therapies. Having strong evidence of gross changes in the volatile metabolome of one line of *APP* mice, we utilized three unique mouse lines which over-express human mutations of the *APP* gene and their respective non-transgenic litter-mates (NTg). Head-space gas chromatography/mass spectrometry (GC/MS) of urinary volatiles uncovered several aberrant chromatographic peak responses. We later employed linear discrimination analysis and found that the GC/MS peak responses provide accurate (>84%) genotype classification of urinary samples. These initial data in animal models show that mutant *APP* gene expression entails a uniquely identifiable urinary odor, which if uncovered in clinical AD populations, may serve as an additional biomarker for the disease.

Alzheimer’s disease (AD), the most common form of dementia, is associated with impaired cognitive, sensory, and motor functions. The pathological hallmarks of AD, namely amyloid-β (Aβ) plaques and neurofibrillary tangles, become more widespread throughout the brain during disease progression^1^. Additionally, both macro- and micro-structural morphological changes occur within the brain during AD progression^2^,^3^,^4^. The common occurrence of these cognitive/behavioral, pathological, and morphological indices throughout AD pathogenesis has resulted in great interest of using these factors, either alone or combined, as biomarkers for the disease.

Biomarkers are critical in the early diagnosis and subsequent treatment of AD^5^,^6^,^7^,^8^. At their most basic level, biomarkers for AD should accurately predict conversion from mild cognitive impairment (MCI) to AD or the identification of people with asymptomatic, though pathologically-definable preclinical AD. To this end, numerous biomarkers for AD are available^5^. For example, cerebrospinal fluid (CSF) Aβ_42_ levels are decreased in individuals with AD (e.g.,^9^), especially those with cerebral Aβ deposition^10^. Brain regions implicated in AD (e.g., the hippocampus) can be imaged *in vivo* and demonstrate progressive reductions in volume when comparing AD to normally aging individuals^2^. Combining these biomarkers allows enhanced diagnostic sensitivity (e.g.,^11^). Explorations to determine novel biomarkers, especially those which are selective for particular pathological features, will be exceptionally valuable in addressing the growing AD epidemic.

One underexplored biomarker source may be found within urine. The chemical composition of urine has been utilized for years to indicate medical disorders (e.g.,^12^,^13^,^14^). Indeed, numerous disorders are characterized by unique volatile odor signatures^15^,^16^, including some lung and breast cancers^17^,^18^, and tuberculosis^19^. Further, aged animals and humans also possess distinct odor phenotypes^20^,^21^. The measurement of urinary F(2)-isoprostanes, markers of free-radical damage^22^, has led to some particularly insightful clues into the basic biology of amyloidosis in mouse models of AD but also to the discovery that urinary levels of this component are greater in transgenic mice overexpressing human mutations of the amyloid precursor protein gene (*APP*), and that elevations in this component precede detectable Aβ plaque load^23^. Thus, urinary composition potentially holds important information regarding pathological status and stage. Furthermore, information regarding an animal’s health status is often communicated via volatile odorants^24^,^25^. Investigation of urinary volatiles as biomarkers of disease has become an active research topic^14^.

We first tested the hypothesis that urinary volatiles would differ between *APP* and NTg mouse urines by using a well-established bioassay designed to identify gross differences in volatile odors. Having demonstrated this difference in one line of *APP* transgenic mice, we utilized three unique lines of *APP* transgenic mice wherein the time-course of pathological progression is well documented, to screen for deviant composition in *APP* mouse urine. We employed gas chromatography-mass spectroscopy (GC/MS) of head space urinary odors from the *APP* transgenic mouse lines to discover putative compounds that vary in concentration among *APP* transgenic mice versus their non-transgenic littermates (NTg). We also utilized linear discrimination modeling to demonstrate that odorant differences in *APP* transgenics can accurately classify samples based upon genotype. Together, these results in animal models of AD pathophysiology support the utility of urinary odor signatures as possible biomarkers of AD.

## Methods

### Subjects

This study used 3 lines of APP transgenic mice and their NTg littermates. All utilized mice were males to reduce variability in urinary composition with the estrous cycle. First, urine samples were collected (as described below) from 15 Tg2576 mice [on the B6SJLF1/J background, generated previously by overexpressing the human KM670/671NL mutation under control of the *PrP* gene^26^] and 17 age-matched NTg littermates ranging in age from 3–16 months. Urine was also collected from 9 TgCRND8 mice [on the B6SJLF1/J background, generated previously by overexpressing the human KM670/671NL + V717F mutation under control of the *PrP* gene^27^] and 9 age-matched NTg littermates ranging in age from 2–8 months. Finally, urine was collected from 10 APPld2 mice [on a bl6/sw background, generated previously by overexpressing the human APP/V717I mutation under control of the *thy1* gene^28^] and 12 age-matched NTg littermates ranging in age from 4–23 months. Age ranges were selected to encompass the span of major amyloidosis within each strain^26^,^27^,^28^. 2–4mo old male bl6 mice were used as ‘sensor’ mice in all behavioral studies, each naïve to each respective test it was used in. The ‘sensor’ mice were naïve to the transgenic strains, having never shared a cage with mice of these strains until the experiment. All mice were bred and maintained within the Nathan S. Kline Institute for Psychiatric Research animal facility and were only used at one age each. Mice were maintained on a 12:12 (light:dark) day cycle in standard plastic cages with corn cob bedding and all urine sample collection and behavioral testing performed during the daylight phase. Mice were genotyped by PCR analysis of tail DNA using standard methods. Experiments were conducted in accordance with the guidelines of the National Institutes of Health and were approved by the Nathan S. Kline Institute’s Institutional Animal Care and Use Committee.

### Urine collection

Urine was collected into odorless microcentrifuge vials from mice (Table 1) as they were held by the nape of their neck^29^. Samples, stored at −80 °C, were collected over the course of > 4 days, until sufficient volume was gathered/mouse. Care was taken to ensure no parts of the subject’s body, nor fecal matter, came into contact with the sample vial. Samples were subsequently thawed and pooled over multiday collections for headspace analysis.

### Odor cross-habituation assay

‘Sensor’ mice were tested for their investigation of volatile odors while in their home cage in an odor cross-habituation behavioral task as described in detail in^30^. Urine collected from Tg2576 and NTg littermate mice (3mo [juvenile] and 12–14mo [aged]) were pooled by donor. To administer the odors to the sensor mice, clean cotton-stick applicators were saturated with ddH20, Tg2576 urine (2–4 or 12–14mo old), or NTg urine (2–4 or 12–14mo old) which was then enclosed in a piece of odorless plastic tubing to prevent contact of the liquid odor with the testing chamber or animal yet still allow volatile odor delivery. Odors (provided fresh on each trial, but from the same donor mouse for each block) were delivered for 4 successive trials (1 block), 20 sec each, separated by 30 sec inter-trial intervals, by inserting the odor stick into a metal vent port on the side of the animal’s home cage. Each presentation (viz., trial) consisted of a homogenized sample from a different individual mouse donor. Increased time spent investigating the first trial of the APP urine sample versus the last trial of NTg urine sample reflects cross-habituation and therefore discrimination^31^,^32^ of the two stimuli based upon their odor. No contact with the stimulus was allowed and thus the behavioral responses are based upon volatile odor, but independent of visual, auditory, gustatory, and/or somatosensory input. Testing took place during the light phase of the animals’ (12:12) day:light cycle, over two daily sessions (3–4 odors/session) separated by 24–48 hrs. The duration of time spent investigating, defined as snout-oriented sniffing within 1 cm of the odor presentation port, was recorded across all trials by a single observer blind to genotypes (D.W.W.). Home cages were cleaned with fresh corn cob bedding 24–48 hrs prior to behavioral testing.

### Headspace Analyses

Analyses were conducted with a HT3 dynamic headspace analyzer (Teledyne Tekmar, Mason, OH, USA) outfitted with Supelco Trap K Vocarb 3000 thermal desorption trap (Sigma-Aldrich Co., St. Louis, MO, USA) attached to a Thermo Scientific ISQ GC-MS equipped with a single quadrapole mass spectrometer (Thermo Scientific, Waltham, MA, USA) and a 30 m × 0.25 mm id Stabiliwax^®^-DA fused-silica capillary column (Restek, Bellefonte, PA, USA). Urine samples were subjected to dynamic headspace GC-MS analysis by placing 25 μL of the thawed urine sample in a sealed 20 mL headspace vial. The vial was maintained at 40 °C, swept with helium for 20 min (flow rate of 75 mL/min), and the volatiles collected on the thermal desorption trap. Trap contents were desorbed at 260 °C directly into the GC-MS using a splitless injection. The GC oven program had an initial temperature of 40 °C (held for 3.0 min) followed by a ramp of 7.0 °C/min to a final temperature of 230 °C (held for 6.0 min). The MS was used in scan mode from 33 to 400 m/z. The chromatographic runtime was 36 min and the scan rate was 3.3 Hz. Urine samples were analyzed in a quasi-random, haphazard order among APP mutation (APPLd2, TgCDRN8, and Tg2576) and genotype (heterozygous or NTg) of donors. In total, there were 72 urine samples obtained from male mice (Table 1). Samples were analyzed in a counterbalanced order over the course of four days and each chromatographic run included several vial “blanks” (empty headspace vials). Peak identifications were assigned on the basis of spectral library search with the NIST Standard Reference Database 1A (US Department of Commerce, Gaithersburg, MD, USA, 2011, Date of access: 01/10/2015).

### Headspace data processing

Chromatographic data were exported to NetCDF format for baseline correction, noise elimination, and peak alignment processing using Metalign software^TM^ 33. Multivariate data resulting from Metalign^TM^ (consisting of all mass spectrometric responses exceeding a defined threshold at each scan event) were then processed using the MSClust tool^34^. MSClust permits unsupervised determination of chromatographic peaks and yields a single response (corresponding to abundance) for each metabolite. The resultant dataset (consisting of 69 peak responses for each of the 72 urine samples) was standardized (mean = 0; standard deviation = 1) and subjected to principal components analysis for visual identification of sample outliers (Supplementary Data 1 and 2). Seven samples were found to have excessive residual variance and were removed from the data set (65 remained). Data sorting was performed by a single experimenter (B.A.K.) who was blind to genotypes.

### Statistical Analyses

Odor habituation behavior data (time in sec) were pooled within age or odor stimuli and analyzed with ANOVA followed by Fisher’s PLSD. The chemometric dataset from 65 samples representing all three strains were subjected to pair-wise comparisons (APP vs. NTg) for each peak response (69 total). Differences were categorized as positive (increased response in APP transgenic) or negative (decreased response in APP transgenic) and the false discovery rate controlling procedure was applied to minimize experiment-wise error associated with multiple pair-wise comparisons^35^. Pair-wise comparisons were repeated three times, once for each APP strain.

Method repeatability was assessed by repeatedly analyzing a composite male C57BL/6 urine sample on multiple analysis days according to the described procedures. The chromatographic responses of acetophenone (an endogenous metabolite) were assessed for within-day repeatability (among three chemical analysis days) and between-days variability. The linear responses of three endogenous compounds (3-methylcyclopentanone, 1-octen-3-ol, and acetophenone) were assessed by fortifying composite urine samples with increasing volumes (10, 20, and 30 μL) of a combined fortification solution of these compounds in water.

Unique linear discriminant analysis (LDA) models were constructed for each of the APP strains. At this point, the experimenter was no longer blind to genotypes as a necessity of constructing the model. Peak responses which significantly contributed to the LDA model were identified using stepwise selection in SAS^®^ (PROC STEPDISC). MSClust-derived peak identifications and original chromatographic data were evaluated to determine if the identified peak(s) were associated with urine samples or artifactual (present in vial blanks). Candidate peak responses were then employed in discriminant analyses using PROC DISCRIM with cross-validation.

## Results

### Behavioral-level evidence for unique volatile odors in APP transgenic mice

**Figure 1:**
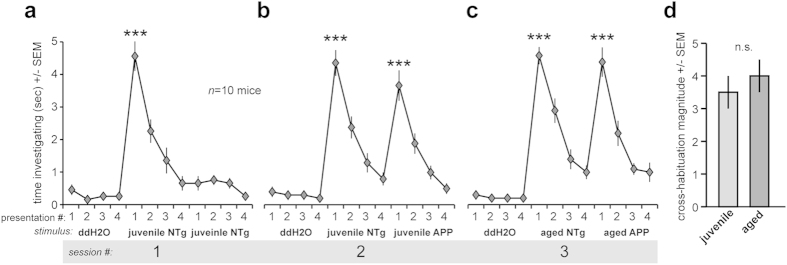
Sensor mice distinguish APP mouse urine from NTg urine. Time spent investigating by bl6 ‘sensor’ mice (*n* = 10) in an odor cross-habituation task. bl6 mice in home cages were acclimated to the task with successive presentations of a cotton stick laced with ddH20, followed by successive presentations of a cotton stick laced with juvenile (2-4mo old) NTg urine (**a**) or separately, juvenile NTg urine followed by juvenile APP (Tg2576) urine (**b**), or in a final separate test, aged (14-16mo old) NTg urine followed by aged APP urine (**c**). ***p < 0.0001, vs. preceding trial #4, ANOVA followed by Fisher’s PLSD. Each presentation consisted of a homogenized sample from an individual mouse. Odor cross-habituation (time spent investigating the first trial of the APP urine sample vs. the last trial of NTg urine sample) was similar (n.s., *p* > 0.05, ANOVA followed by Fisher’s PLSD) between juvenile and aged mice (**d**).

We sought to demonstrate that volatile urinary odor composition, specifically, is altered in APP transgenic mice. As an initial behavioral test for this, individual mice were presented with a series of successive stimuli in an odor cross-habituation task (see Methods). As shown in Fig. 1A, following presentation with ddH_2_0, NTg ‘sensor’ mice showed minimal cross-habituation to urine from juvenile (2–4mo of age) NTg mice upon the first presentation (*F*(1,18) 82.791, *p* < 0.0001) and displayed a subsequent habituation to successive presentations of juvenile NTg littermate stimuli. When presented with urine from a second NTg juvenile, the sensor mice showed strong cross-habituation, suggesting minimal difference in urinary odor between these NTg mice. In contrast and supporting our hypothesis for specific urinary odor compositions in APP mice, following habituation to successive presentations of age-matched NTg urine (Fig. 1B,C), sensor mice showed no cross-habituation to juvenile (*F*(1,18) 31.937, *p* < 0.000 1) (Fig. 1B) and aged (14–16mo of age) Tg2576 mouse urine (*F*(1,18) 46.446, *p* < 0.0001) (Fig. 1C), suggesting detectable differences between the urinary odors of NTg and APP mice. The magnitude of cross-habituation between NTg and Tg2576 mouse urine was similar regardless of sample ages (Fig. 1D) (*F*(1,18) 0.506, *p* = 0.486), suggesting that urinary odor from juvenile mice (at the most early stages of pathogenesis^26^,^30^,^36^,^37^) is as readily discriminable as urinary odor from aged Tg2576 mice with more severe cerebral pathology.

### Specific urinary chemical differences among APP transgenic mouse lines

Evaluation of acetophenone peak responses demonstrated that method variability was excellent. The overall relative standard deviation (RSD) for 21 analyses was 8.67%. Further examination indicated that within-day repeatability was 6.58% and between-day variability was 7.14%. Responses of the fortification compounds increased linearly with coefficients of determination (r-square) of 0.993 for 3-methylcyclopentanone, 0.962 (1-octen-3-ol), and 0.991 (acetophenone).

**Figure 2:**
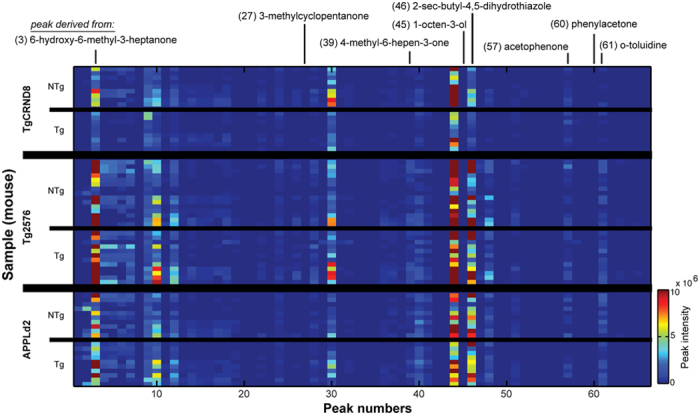
Distinct GC/MS peaks in APP mouse urine. 2-dimensional histograms of head-space gas chromatography and mass spectroscopy (GC/MS) results from NTg and APP male mice, across three strains. Y-axis bins = individual mouse results. Data are scaled similarly across all strains and genotypes. Data along the z-axis represent peak areas generated in MSClust (see Materials and Methods). Location of some notable compounds are indicated (also see Table 4).

We sought to employ a metabolomic approach to identify chemical profile differences between APP and NTg mouse urinary odor by means of headspace GC/MS. To provide a comprehensive, strain- and promoter- independent assessment of these differences, we utilized three unique strains of APP transgenic mice (Tg2576^26^, TgCRND8^27^, APPLd2^28^) and their respective NTg littermates, all at a range of ages (Table 1). We observed 69 unique chromatographic peaks tentatively identified by their mass spectra (Fig. 2) (Supplementary Data 1 and 2). Of these, phenylacetone significantly increased in APP transgenic mice across all strains (*p* = 0.0044). Examinations among individual strains identified decreased responses (relative to analogous NTg) of 3-methylcyclopentanone (*p* = 0.0024), 4-methyl-6-hepten-3-one (p = 0.004); and a cyclic enol ether (*p* = 0.0044) representing the known mouse pheromone 6-hydroxy-6-methyl-3-heptanone^38^ in TgCRND8 mice (Table 2). An increased phenylacetone response in APP mice (*p* = 0.001) was the only observed difference within the Tg2576 strain. No pair-wise differences were found when data from the APPLd2 strain were subjected to strain-specific analysis.

### Predictive classification of APP genotypes based upon GC/MS peaks

We utilized peak headspace compound responses from individual mice (pooled within genotypes and strains) to create linear discriminant analysis (LDA) models. Unique models were created for each strain consisting of only two (TgCRND8), three (APPLd2), and four (Tg2576) chemical variables (Table 2). Interestingly, one compound, 1-octen-3-ol, was an important variable in all three models. LDA results demonstrated excellent classification success with good cross-validation error rates (<16%) for all three strains, despite being derived from urine samples collected from mice ranging in age from 2 to 23 months (Table 3). In summary, volatile odor signatures from APP transgenic mouse urine differ uniquely from NTg littermates and when combined with discriminatory analysis, allow for the classification of APP vs. NTg genotypes.

## Discussion

In the present study we sought to determine whether APP transgenic mice possess unique volatile urinary chemical differences. Genetic-induced changes in body odor composition are established in some models of pathology (e.g.,^39^), but not in the AD literature. We first addressed the hypothesis that volatile metabolites are altered in APP transgenic mice by utilizing a highly sensitive, yet non-descriptive rodent behavioral assay. Previous use of the odor cross-habituation assay has shown that animals habituate across trials with the same animals scent^40^ and dishabituate to inter-animal differences in odor^41^. The use of odor-guided behavior as an initial test of volatile odor-differences in APP transgenic mice in the present report has several advantages. First, rodents possess a highly-acute sense of smell, being able to detect low concentrations of odors^42^ and discriminate between fine differences in stimuli^43^,^44^. Further, rodents display a high level of motivation to seek out and investigate odors^45^,^46^,^47^. Our initial hypothesis that mice are able to discriminate differences between conspecifics based upon APP gene expression, as reflected in odor cross-habituation test results, suggests a potent odor signal present in APP transgenic mice.

Having strong evidence that the volatile metabolome differed between *APP* and NTg mouse urines, we undertook a comprehensive metabolomic examination of urinary odorants by employing three unique APP lines all associated with progressive elevations in Aβ and other APP metabolites^26^,^27^,^28^ to identify specific compounds which are altered in concentration within the context of this over-expressed mutation. Examination of relative concentrations of volatiles (i.e. metabolomics) has become a common practice for identifying metabolic profiles, or “fingerprints” in both plant^48^ and animal^49^ systems. Furthermore, the Metalign™ tool utilized in this work has been demonstrated to be one of the highest performers for detecting true positive metabolites among the many available metabolomic tools^50^,^51^.

By employing a metabolomic approach, we found chemical evidence for unique volatile odor signatures in APP transgenic mouse models, thereby suggesting the clinical utility of volatile odor signatures as possible biomarkers for AD. The confirmatory results of the two independent methods for examining urinary volatiles do not similarly certify that specific odorants of the chemometric models and the compounds giving rise to dishabituation in behavioral assays are synonymous. Furthermore, it is important to highlight that numerous differences exist between human and murine metabolism^52^ and thus these results are intended to be proof-of-concept and not for immediate applicability towards clinical studies.

Our GC/MS experiments demonstrate that patterns of urine volatiles are altered in a predictable manner in APP transgenic mice. This genotype-effect was conserved across the three different lines of APP mice, albeit attributable to largely different components. Genetic alteration of urine volatiles did not include the appearance of novel compounds detectable by GC/MS methods, but instead elevations or reductions in their respective concentrations. In fact, all peaks useful for genotype discrimination were present in both APP and NTg individuals. Furthermore, these compounds have been previously identified in mouse urine and implicated in a variety of biological processes, such as maturation, diurnal variability, stress^53^, cancer biology^54^, and/or immunological genotypes^55^ (Table 4). Among the eight compounds utilized in the LDA models (Table 2), two were particularly notable. These compounds are phenylacetone (increased concentration in APP transgenic mice) and 1-octen-3-ol (contributing to all three LDA models). Recognized as a metabolite of amphetamine, phenylacetone has been regularly identified in mouse urine^53^,^55^,^56^,^57^,^58^. Particularly, in mice, phenylacetone concentrations increase during periods of activity (p.m., dark phase cycle) and decrease with maturation^53^. Thus, the finding of elevated phenylacetone among both juvenile and aged APP mice in this study indicate a metabolic abnormality throughout the life span in these mice, consistent with altered metabolic properties in persons with AD (e.g.,^59^). The route mechanism of increased phenylacetone concentration in all APP strains studied here, might include disruption of phenylalanine metabolism, and likely will hold important clues to understanding common pathological conditions in all three models.

Urinary concentration of 1-octen-3-ol is under genetic control in mice and is impacted by alterations of diet, but not the interaction of these effects^55^. 1-octen-3-ol has been identified as a biomarker of human *Campylobacter jejuni* infection in feces^60^ and a biomarker for liver cancer in human blood^61^. Though commonly considered to be produced by enzymatic oxidation of C18-polyunsaturated fatty acids in fungi, Xue *et al*. speculate that 1-octen-3-ol may be an intermediate or end-product produced by mammals for its antioxidant activity to reduce concentrations of carcinogenic substances^61^. Thus, while both elevated compounds may have links to AD pathogenesis, it is presently unclear why the magnitude of responses from these and the other compounds were altered in these mouse models. Indeed, the mechanisms whereby mutant APP expression may induce urinary changes are largely unknown.

Classification of APP genotypes was not biased by the age of the mouse urine donor as evidenced by the low cross-validation error rates of urines collected from a range of donor ages (Table 3). Our finding that age did not appear to interfere with the statistical models suggests that composition differences between APP and NTg mice is mostly governed by APP gene expression. These three transgenic APP mouse strains all accumulate Aβ, other APP metabolites, and display additional pathological hallmarks (e.g., microglial activation) throughout aging (albeit at differing time-points). In part due to this, we selected to pool samples across-ages, yet within strain and genotype to assess genotype effects. Future longitudinal studies, analyzing within-subjects changes in urinary samples with age, might provide additional information on the changes in urinary odor composition throughout pathogenesis. Given the progressive elevations in Aβ displayed by all three stains of APP mice used herein, it is also perhaps surprising that we found respectfully narrow alterations in urinary odor composition which were shared across all three stains of APP mice. While within each APP strain multiple compounds were found to significantly deviate in concentration from that of NTg mice, only one compound (1-octen-3-ol) was significantly altered in concentration across all three strains. This might be explained by differences in APP gene dosage in the differing strains of mice and/or the specific APP mutation expressed. It may also be possible that the APP transgene, independent of Alzheimer’s disease, influences the expression of urinary chemicals by means of random chromosomal insertion and/or the number of copies. Inclusion of even more AD model mouse strains (i.e., harboring over-expressions of the Swedish mutation) may help understand this and also allow for tests of whether the volatile metabolome differences observed herein may be differently present in pathologically burdened, versus, spared mice.

In conclusion, our findings in mouse models of AD suggest that volatile odor signatures are also likely to be observed in human AD populations and may be informative early indicators of AD during prodromal disease states. An arguably ideal test of this could be performed in human ApoE4 (apolipoprotein E type 4 allele) positive populations compared to ApoE4 negative. This would have to be carefully performed while controlling for fluctuations in dietary intake, hormone levels, other medical disorders, and/or medical treatment which could each impact urinary odor composition. We predict that future work incorporating volatile urinary odor quantification concurrent with other more standardized biomarkers, including CSF Aβ, brain volume measures, PiB imaging, and cognitive testing will be essential in translating the efficacy of this finding into a sensitive clinical diagnostic.

## Additional Information

**How to cite this article**: Kimball, B. A. *et al*. Alterations of the volatile metabolome in mouse models of Alzheimer’s disease. *Sci. Rep.* **6**, 19495; doi: 10.1038/srep19495 (2016).

## Supplementary Material

Supplementary Dataset 1

Supplementary Dataset 2

## Figures and Tables

**Table 1:** Number of male mouse urine samples subjected to chemical analyses and age range of donors (months).

	APPLd2 (4–23 mo.)	CRND8 (2–8 mo.)	Tg2576 (3–16 mo.)	Total
Wild-type	12	9	17	38
Heterozygous	10	9	15	34
Total	22	18	32	72

**Table 2:** Participation of relevant urine volatiles in statistical models.

Compound	Pair-wise Result^1^	LDA Model^2^		
		APPLd2	TgCRND8	Tg2576
6-hydroxy-6-methyl-3-heptanone^3^	Decrease (TgCRND8)			
3-methylcyclopentanone	Decrease (TgCRND8)	**x**		
4-methyl-6-hepten-3-one	Decrease (TgCRND8)		**x**	
1-octen-3-ol		**x**	**x**	**x**
2-*sec*-butyl-4,5-dihydrothiazole				**x**
acetophenone				**x**
phenylacetone	Increase (Tg2576)			**x**
*o*-toluidine		**x**		

^1^Change in concentration of amyloid precursor protein transgenic mouse relative to non-transgenic wild-type mouse (specific strain provided in parentheses).

^2^Linear discriminant analysis (LDA) model by strain.

^3^Identified as cyclic enol-ether chromatographic artifact^38^.

**Table 3:** Linear discriminant analysis (LDA) results for models developed from urine volatiles for each strain.

Strain	Model Summary^1^		Model error rate	Cross-validation error rate
	APP	NTg		
APPLd2	10 of 10	8 of 10	10%	15%
TgCRND8	9 of 9	7 of 9	11%	11%
Tg2576	10 of 12	15 of 15	8.3%	15.8%

^1^Rate of correct classification of individual urine donor as amyloid precursor protein transgenic (APP) or non-transgenic wild-type (NTg).

**Table 4:** Relevant urine volatiles and their previously reported roles in murine biology.

Compound	Age^a^	Diurnal^a^	Stress^a^	Cancer^b^	MHC^**c**^	Diet^c^	Reproductive Pheromone^d^
6-hydroxy-6-methyl-3-heptanone^1^				—2			**x**
3-methylcyclopentanone					**x**^3^	**x**^4^	
4-methyl-6-hepten-3-one						**x**	
1-octen-3-ol					**x**	**x**	
2-*sec*-butyl-4,5-dihydrothiazole	+^5^		—6	—	**x**	**x**	**x**
acetophenone		—7					
phenylacetone	—	+					
*o*-toluidine				—		**x**	

^a^Schaefer *et al*., 2010 53.

^b^Matsumura *et al*., 2010 54.

^c^Kwak *et al*., 2008 55.

^d^Timm *et al*., 2001 62.

^1^Identified as cyclic enol-ether chromatographic artifact 38.

^2^Concentration (+increase; −decrease) relative to presence of lung tumor.

^3^Concentration differed between two major histocompatibility complex (MHC) types.

^4^Concentration differed between two distinct diets.

^5^Concentration (+increase; −decrease) relative to 8 week versus 4 week age.

^6^Concentration (+increase; −decrease) relative to presence of restraint.

^7^Concentration (+increase; −decrease) relative to pm versus am.
